# On the architecture of cell regulation networks

**DOI:** 10.1186/1752-0509-5-37

**Published:** 2011-03-02

**Authors:** Yueheng Lan, Igor Mezić

**Affiliations:** 1 Department of Physics, Tsinghua University, Beijing 100084, China; 2 The Center for Control, Dynamical Systems and Computation, University of California, Santa Barbara, CA 93106, USA; 3 Department of Mechanical Engineering, University of California, Santa Barbara, CA 93106, USA

## Abstract

**Background:**

With the rapid development of high-throughput experiments, detecting functional modules has become increasingly important in analyzing biological networks. However, the growing size and complexity of these networks preclude structural breaking in terms of simplest units. We propose a novel graph theoretic decomposition scheme combined with dynamics consideration for probing the architecture of complex biological networks.

**Results:**

Our approach allows us to identify two structurally important components: the "minimal production unit"(MPU) which responds quickly and robustly to external signals, and the feedback controllers which adjust the output of the MPU to desired values usually at a larger time scale. The successful application of our technique to several of the most common cell regulation networks indicates that such architectural feature could be universal. Detailed illustration and discussion are made to explain the network structures and how they are tied to biological functions.

**Conclusions:**

The proposed scheme may be potentially applied to various large-scale cell regulation networks to identify functional modules that play essential roles and thus provide handles for analyzing and understanding cell activity from basic biochemical processes.

## Background

Cellular behavior, including motility, metabolism and reproduction is controlled by complex biochemical reaction networks, many of which have been identified and studied in detail [1]. These networks realize their regulatory roles through complex molecular interactions. Contemporary high throughput experiments produce unprecedented amount of data that serve to pinpoint the players and their interactions, resulting in complex chemical reaction graphs. How to analyze these intricate graphs and gain insight into the regulation mechanism employed by cell has become a central problem of molecular biology.

Much progress has been made in the analysis of functions of complex networks, no matter if they are modeled deterministically [2,3] or stochastically [4-9]. These studies concentrate on the investigation of dynamics of given networks by checking their stability, parameter dependence, robustness and input-output relation. However, for large-scale networks such as those commonly found in important biological processes [10,11], the incurred computational load often severely limits our ability for performing detailed analysis. More critically, with continued experimental efforts that are revealing more details of networks' global wiring, their growing complexity has made it harder and harder to identify the underlying local functional structures and thus probe the network function.

Normal cell life involves physical or chemical activities at vast range of spatial and temporal scales and it is vital to identify characteristic structures at all scales and study their roles in relation to a particular cell function [12-17]. These key structures are called modules, the existence of which contributes almost to every aspect of the cell regulation: robustness, sensitivity, adaptivity, evolvability. Their detection and study much simplifies the analysis of complex networks since a small set of modules could come from and be a lot simpler than a collection of many entangled individual agents [18]. The simplification may be carried on by constructing modules of modules.

Recently, useful concepts distilled from statistical physics such as the small-world and the scale-free networks [19,20], began to see their application in gene regulation networks and lead to considerable success in unraveling the statistical nature of these networks. However, this type of statistical analysis mainly aims at gross features of networks [21] and thus ignores local structural properties and heterogeneities, which often determine the operation of a network in an essential way, since disparate network modules generally imply distinct dynamics and fit for different functional requirements [22,23]. Nevertheless, the determination of modular structure in a large network is not straightforward since one molecular species may be involved in many different pathways with very distinct external connections. Such inter-correlation is easily under-appreciated and yet has profound consequences on the organism.

In this paper we propose a new theory of architecture of biochemical networks based on control and graph theoretic analysis. In this theory, a network consists of two major modules: one is the pipeline of linear information production unit which serves to generate the required output (e.g. protein concentrations); the other is the set of feedback loops which act as controllers of the production. These two modules are identified based on the information flow in a network. Specifically, input and output nodes define a polarity of the network. Information is received at the input, processed and then sent to the output. The agents that carry on the information along the forward direction belong to the production unit. The remaining agents direct part of the information in the opposite direction and thus are elements of the feedback controller [22]. In the paper, detailed algorithm are presented for the construction of the production unit and the feedback controller.

The concept of modules has been used in modeling of biological networks for decades. The existence of this special structure is universally agreed upon but its exact definition is done on case-by-case basis. Recently, modules and community structures are defined in the graph theoretic studies of many real-world networks [20,24], based on the connectivity between nodes. Useful as it is, this type of definitions ignore the importance of controller loops. The community structure in the synchronization study involves more dynamics information but it works for a special class of networks and for particular types of equations of motion. Closely related concepts, such as "network motif" are also proposed [13,25]. Motifs consist of a small number of nodes and appear repeatedly (more than expected from pure statistical consideration) in a network. The modules determined by our algorithm are different from all these in that we emphasize the information processing and controlling units but not simple fixed graph structures given a priori. In contrast, the decomposition procedure based on the function of the network and the associated polarity supplies the detailed structures of our modules. Different polarities may result in different decompositions and different initial conditions may define different MPUs. So our concept of modules depends on the information flow through or the function of the network.

In the following, we will use the NF*κ*B regulation network [26] as an example to explain our graph theoretic analysis procedure and display the generic producer-controller structure. We also analyze the chemotaxis network of *E. coli*, TNF-*α *initiated apoptosis network [27], the circadian clock network in *Drosophila *[28] as interesting examples of the proposed architecture. Three more examples of biological networks are presented in Additional File 1 and are all found to possess the same architecture.

## Results and Discussion

### The NFκB regulatory network

The NF*κ*B regulatory pathway concerns the switching dynamics of the nuclear factor NF*κ*B, which regulates various genes important for pathogen or cytokine inflammation, immune response, cell proliferation and survival [29,30]. In the cytoplasm of a resting cell, NF*κ*B usually binds to I*κ*B*α *and its activity is suppressed. Certain external signals activate the switch protein IKK which phosphorylates I*κ*B*α *such that NF*κ*B is released [31]. The free NF*κ*B then translocates into the nucleus and initiates the transcription of a large set of proteins, including protein I*κ*B*α *and protein A20. Protein I*κ*B*α*, once synthesized in the cytoplasm, enters the nucleus, binds to NF*κ*B, transports it out to the cytoplasm and thus terminates the transcription. Protein A20 deactivates IKK. Therefore, the module mainly consists of two forward proteins IKK and NF*κ*B and two feedback proteins I*κ*B*α *and A20. Also, the translocation of the proteins between the nucleus and the cytoplasm is an important biological process that realizes spatial localization of different protein species.

**Figure 1:**
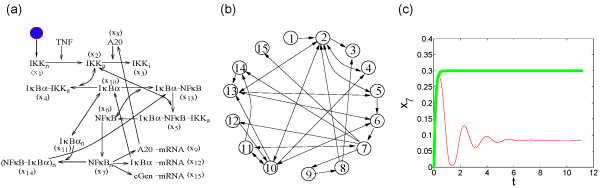
**A model of the NF-***κ***B signaling module**. (A) The structure diagram of the NF-*κ*B module. (B) The derived interaction graph. (C) The time evolution of the NF*κ*B*_n _*with the full network (red line) and with the minimal production unit (MPU) (green line).

The diagram of a detailed model of the NF*κ*B regulatory network is shown in Figure 1A where we use *x_i_*'s to represent the concentration of various proteins. The associated chemical kinetic model is given and explained in Additional File 1. With physiological initial conditions [32], the concentration of the nuclear NF*κ*B executes damped oscillations, shown with the thin dotted curve in Figure 1C. At the beginning, it shoots up to a very high value in a short time and then relaxes to a much lower steady value in an oscillatory way.

For any networked system described by certain dynamical equations, it is easy to write an interaction graph with the vertices representing the reacting agents and the edges directed from each agent to the ones under its influence. The interaction graph for the NF*κ*B model is shown in Figure 1B.

It is straightforward to write down the adjacency matrix for the interaction graph, which marks 1 at the entries corresponding to connected edges and zero otherwise. The interaction graph and the adjacency matrix neglect details of the interactions and only map out the network topology which holds true almost everywhere in the phase space and the parameter space, except for a set of measure zero [33]. This robustness confers flexibility of analysis to analyzing vastly different dynamics described by ODEs or mappings or even stochastic equations. Certain system properties, like the uniqueness of the stationary point sometimes can be deduced from pure topological consideration of network structures [34,35]. So, understanding of structure of interaction graphs helps unveil the key elements in a complex system which possibly has uncertainties in the parameter values or is influenced by a noisy environment. Graph theoretic techniques will be developed here to enable an automatic decomposition of a biochemical network into forward and feedback modules, thus unraveling the architecture responsible for its biological function.

### Controllers of the NFκB network

The horizontal-vertical decomposition (HVD) of an interaction graph of a dynamical system has been discussed in a paper [33]. It is a technique that studies information flow and processing in interconnected systems. Vertically, the HVD decomposes a system into a linear series of layers, where the layer downstream is influenced by the layer upstream but not vice versa. So, the input signal propagates unidirectionally. Horizontally, the HVD decomposes each layer into independent groups with no direct connections between. In one layer, each group receives its own input from upstream layers and output the signal to downstream layers. Each group is a strongly connected component (SCC) such that a path always exists between any two nodes in the group. If each group collapsed into a point, the whole network will become cycle-free [36].

Direct application of the HVD to the interaction graph in Figure 1B results in three layers with the top and bottom layer consist of the vertex sets {*x*_1_}(IKK*_n_*) and {*x*_3_, *x*_15_}(IKK*_i_*, cGen-mRNA), respectively. The rest of the vertices are strongly connected and belong to the middle layer. This type of structure with dominant intermediate processing unit exists in most biological and engineering networks [33,37] as a result of omnipresent feedback loops and reversibility of many biochemical reactions. Below, we apply our cycle search and selection technique to the middle layer for further decomposition into the production unit and feedback controller.

**Figure 2:**
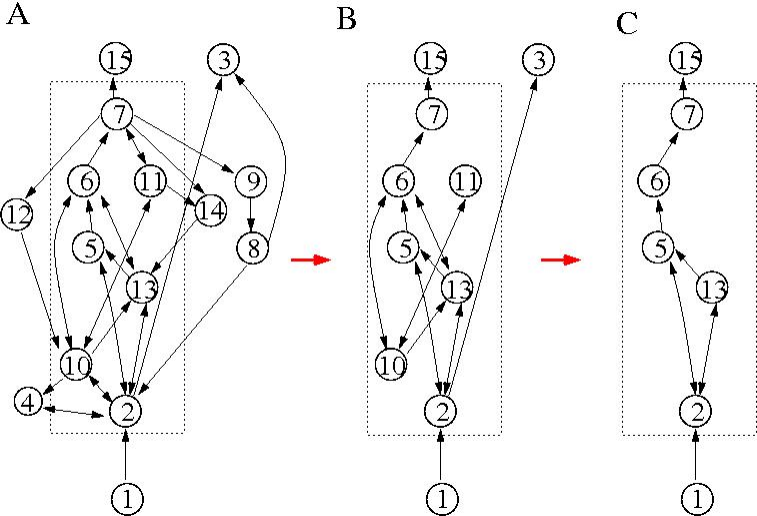
**The structure decomposition and the MPU of the NF-***κ***B signaling regulatory network**. (A) The structured diagram derived from the graph theoretic analysis; (B) with feedbacks removed. (C) The MPU with irrelevant vertices removed.

The polarity of the middle layer is ready to be identified. The vertex *x*_15 _is the output signal that is of interest while *x*_1 _receives the external input. Therefore, in the middle layer, *x*_2 _is the input vertex and *x*_7 _is the output one. In the mean time, we observe that in an SCC, if feedbacks exist, they are always making cycles and vice versa every cycle contains at least one forward and one feedback edge. As cycles are obvious topological invariants of a network and easy to seek, our strategy consists of two steps: first, search for all cycles that exist in the graph; second, determine the feedbacks through a selection procedure, which depends on the polarity of the network. The detailed illustration of our technique is contained in the Methods section. Here we show the computation result in Figure 2A, where we see that our procedure identified four feedback loops:

• FB*_a _*- the one through vertex 4: IKKa associates with free I*κ*B*α *and catalyzes its decay.

• FB*_b _*- the one through vertex 14: I*κ*B*α_n _*captures NF*κ*B*_n _*to form (I*κ*B*α*-NF*κ*B)*_n_*, which then moves out of the nucleus.

• FB*_c _*- the one through vertex 12: NF*κ*B*_n _*promotes the production of the I*κ*B*α *mRNA which translocates to the cytoplasm and initiates a burst of I*κ*B*α *production.

• FB*_d _*- the one through vertices 8 and 9: NF*κ*B*_n _*promotes the production of the A20 mRNA and thus initiates the production of A20, which catalyzes the decay of IKK*_a_*.

This identification agrees very well with the usual recognition of feedback loops of this system in the literature [29,30] based on biological reasoning. The correct identification of feedback loops is essential for understanding the signal processing of a network since many important cellular activities are controlled or even realized by feedback signaling [22,23]. We emphasize that we recognized the feedback loops by an automatic procedure based on graph decomposition.

### Extracting the minimal production unit

After the structured network is constructed as in Figure 2A, we proceed to the extraction of the minimal production unit (MPU). In the case of signal transduction network, the MPU is the minimal subgraph of a network that produces a response to external stimuli. The MPU is minimal in the sense that removal of any links from the subnetwork will lead to zero output. However, the response of the MPU may happen at a value that is different from what is desired in a real cell and setting that correct value is one of the roles of the feedbacks. Its identification depends both on the initial state of the system and on the signal that is of interest. Moreover, certain qualitative aspects of chemical kinetics of the network need to be considered in the course. As a matter of fact, the binary or dissociative reactions correlate certain edges that represent same reactions. For example, the associative reaction *A *+ *B*→ *C *is depicted as *A→ C *← *B *in the interaction graph and the two arrows represent the same reaction. In previous computation, we ignored this correlation and carried out our analysis purely from a graph theoretic point of view. A more detailed consideration needs to incorporate this correlation: these two arrows have to coexist. Below, the NF*κ*B network is used as an example to demonstrate the procedure of the MPU extraction in detail.

As we now only consider the forward production part to output *x*_15_, the feedbacks and the associated reactions are first removed. For the NF*κ*B network, we remove {*x*_4_, *x*_8_, *x*_9_, *x*_12_, *x*_14_} and arrive at Figure 2B. The correlation among edges has been considered as suggested by the above-mentioned binary reaction, i.e., the correlated arrows will be removed or kept coincidentally. Next, all the outputs except the one we are interested in are removed. That is, {*x*_3_, *x*_11_} are removed. Here we see that the final MPU indeed depends on what signal we are looking at. Different output may result in different MPUs. Finally, we remove other irrelevant vertices in a recursive way according to the topology of the resulting graph and the given initial conditions. In the NF*κ*B example, based on Figure 2B, *x*_10 _is removable since it does not lie on the main information path and *x*_10_(*t*) = 0 all the time with *x*_10_(0) = 0 being given. All this being done, we produce the MPU depicted in Figure 2C.

The MPU of the NF*κ*B network contains the vertex set *S_m _*= {*x*_1_, *x*_2_, *x*_5_, *x*_6_, *x*_7_, *x*_13_, *x*_14_}, while all other vertices can be regarded as functional controllers. To check if what we got in Figure 2C is indeed an MPU, we keep only the variables in the vertex set *S_m _*and their interactions in the evolution equation. Numerical simulation of this reduced set of equations produced an output curve depicted with the thick solid line in Figure 1C, which displays a fast approach to a steady state value that is much larger than the equilibrium value of the full system. It is interesting to note that the saturation value and the relaxation time are very close to those of the first oscillation peak of the full equation. The vertex set *S_m _*constitutes the MPU of the NF*κ*B gene regulation network, and it is the smallest subgraph that generates a quick and large response to the external signal. It can be checked that cutting any link in Figure 2C will totally disrupt the output-producing ability. For example, if the edge (2, 5) (from *x*_2 _to *x*_5_) is cut, the edge (2, 13) has to be cut as well because of the correlation mentioned earlier, and there will be no output signal. The vertices non in *S_m _*act as controllers to bring down the initial pulse to a desired steady value in a larger time scale. Both the short and the long time response in this network bear important biological significance [30].

### Biological significance of the MPU and the feedbacks

So far, we have identified the MPU and the feedbacks. Next, we go on to discuss the biological relevance of these "modules" to the operation of NF*κ*B network. In this and several other networks we studied, as an important observation, we find out that the MPU is the core signal production unit which responds quickly to the external cues. In the NF*κ*B network, when a signal such as TNF arrives, IKK*_n _*gets immediately activated into IKK*_a _*while the deactivation of IKK*_a _*is minimized since its constitutive decay rate is small. So, the concentration of IKK*_a _*will rapidly increase until A20 is produced by the feedback loop and starts the catalyzed decay of IKK*_a_*. The forward reaction rate is thus maximized transiently and enables cell response to signals with short duration [30]. So, the network has a very sensitive and fast transient response, which is essential for certain signaling pathways [30].

The feedback structures we identified respond at a much larger time scale. Only when the concentration of NF*κ*B reaches a high enough value and induces significant transcriptions in the nucleus, does the negative feedback start to bring down the IKK*_a _*concentration to a steady level which is much lower than the transient peak. The feedback FB*_b _*mainly facilitates the step of clearing NF*κ*B out of the nucleus. FB*_c _*is to restore the concentration of I*κ*B*α *that has been consumed by the IKK*_a_*-catalyzed decay. FB*_d _*is to deactivate IKK*_a _*by *A*20 to bring down the activation level of the whole network. Thus, our structural decomposition detects forward production unit for quick reaction and feedbacks responsible for long time responses.

Like other feedback signaling from the output [4,38], these loops bring about sensitivity and robustness to the network for fulfilling its basic function [39]. The oscillation observed in Figure 1C is a signature of trading stability for sensitivity [17]. The forward immediate amplification confers easy excitability to the network while together with the delayed feedbacks brings about oscillations. On the other hand, over long time, the reaction rates of all biochemical processes are to some extent influenced by environmental variables such as temperatures, pH values, concentrations of certain ions [40]. To function normally under different conditions, the chemical network should possess structural stability. Here the double feedbacks FB*_c _*and FB*_d _*offer extra structural stability against parameter uncertainty: if the parameter changes incur a temporary increase of the concentration of NF*κ*B*_n_*, then both FB*_c _*and FB*_d _*will act to bring it down. Even if one of FB*_c _*or FB*_d _*does not function well, the other one will minimize the change of NF*κ*B concentration. Computation shows that when the rate of the reaction involving either FB*_c _*or FB*_d _*assumes 50% of their normal value, the output signal changes little. However, major changes in the oscillation period, amplitude and the final equilibrium value of the output *x*_7 _are observed when both of the previous changes are made simultaneously. Therefore, these feedbacks provide extra protections for keeping the system stable under parameter fluctuations [22].

The above procedure of searching for MPU is easily generalized to more complex networks, with possible multiple inputs and outputs which interact with each other. We will study their competition or cooperation all together instead of individually. The critical step lies in our capability of detecting feedback loops. Once the feedback controllers are found, the MPU is obtained by removing all the feedbacks and then all the dynamically inessential nodes. The observed separation of time scales, can, however, leads to further theoretical study using averaging methods or normally hyperbolic invariant manifold concept from dynamical systems. We expect to pursue this in our future studies. In what follows, we analyze the *E. coli *chemotaxis network and several other signaling networks. More examples are available online in the Additional File 1.

### Decomposing the E. coli chemotaxis network

**Figure 3:**
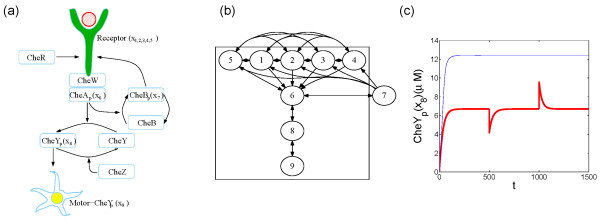
**The chemotaxis model of E. coli**. (A) The structured biochemical diagram. (B) Feedback and forward structure through graph decomposition. (C) The response of CheY*_p _*to the external cue of the full network (thick solid line) and the MPU (thin solid line), ligands added at *t *= 500 and removed at *t *= 1000.

Figure 3A displays a chemotaxis model of *E. coli *[41], which enables the *E. coli *cell swimming to food sources and away from hostile environments. The most salient feature of the chemotaxis regulation network is the sensitivity and adaptivity. That is, *E. coli *is able to respond quickly to very weak signals - concentration gradients and under vastly different background concentrations. This special dynamical properties are insured by interesting network topology [4]. As shown in Figure 3A, chemoattractants (indicated by the red ball) bind to and activate the transmembrane receptors ({*x*_1_, *x*_2_, *x*_3_, *x*_4_, *x*_5_}), which stimulate CheA (*x*_6_) through the adaptor CheW. Activated CheA phosphorylates CheY(*x*_8_), which binds to the flagellar motor (*x*_9_) and increases the frequency of *E. coli *tumbling. The activation of the receptor complex is controlled by its methylation states. Higher methylation states indicate higher probability to be activated. In the model, CheR binds only to the inactive receptors to increase methylation and phosphorylated CheB(*x*_7_) only to the active receptors to decrease methylation.

Figure 3B displays its feedback and forward structure upon application of graph decomposition. The first level consists of the vertex set {*x*_1_, *x*_2_, *x*_3_, *x*_4_, *x*_5_} which are different methylation states of the receptor complex. External signals propagate down through *x*_6_, *x*_8 _and finally reaches the flagellar protein *x*_9_.

There is one feedback vertex *x*_7 _(CheB*_p_*). The minimal production unit (MPU) is obtained after all the reactions involving *x*_7 _are removed and is contained in the box in Figure 3B.

With the feedback through CheB*_p _*(*x*_7_), the system has sensitive detection and robust adaptivity as shown with thick solid line in Figure 3C. Starting with zero value, the CheY*_p _*quickly reaches the saturation level. At *t *= 500*s*, an external stimulus - 10*μM *concentration ligand is supplied, which induces a drop of CheY*_p _*concentration followed by an exponential decay back to the saturation value. At *t *= 1000*s*, the ligand is removed which triggers a jump of CheY*_p _*concentration but regains its stable value exponentially fast. When the feedback is removed, the MPU reaches the stable value after a quick initial rise and stays at the value no matter how the concentration of external ligand changes. The robustness is retained but the adaptivity is lost. So, in this example the feedback is essential for the system's transient response to external stimulus and maintaining the adaptivity. As in the previous example, the production-controller dichotomy structure guarantees the normal functioning of a cell regulation network with both parts playing irreplaceable roles. Here, the forward production reacts quickly accounting for the sensitivity of the network while the controller works in a larger time span to realize the adaptivity.

### Survival and apoptotic pathways initiated by TNF-α

**Figure 4:**
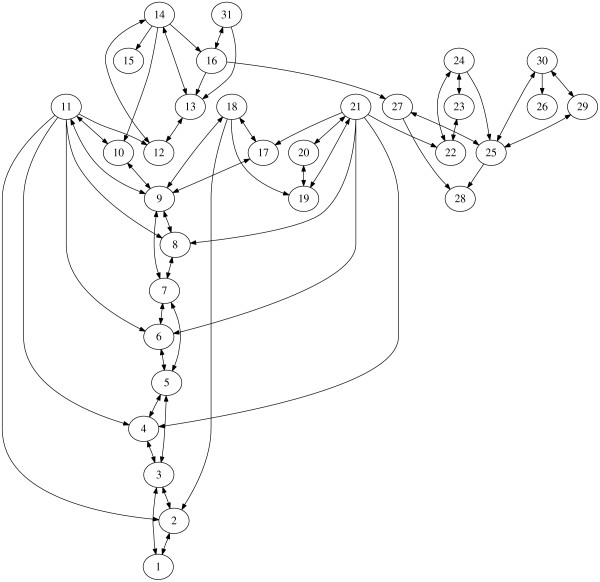
**The TNF***α ***model**. Network representation of the Survival and apoptotic pathways initiated by TNF-*α*.

This model studies the survival and apoptotic pathways initiated by TNF-*α *that we adopt from [42]. These pathways play decisive roles in cell fate decision in response to inflammation and infection. After an external cue TNF-*α *binds to its receptor TNFR1 (*x*_2_) (see the table), adaptor proteins TRADD, TRAF2 and RIP-1 are recruited to form an early complex ready for binding and activating other functional proteins. There are two different downstream pathways: the survival pathway mediated by NF-*κ*B and the apoptotic pathway mediated by caspase. NF-*κ*B is usually sequestered by I*κ*B and is released when I*κ*B degrades. IKK binds to the early complex to form a survival complex and is activated with the dissociation of this complex. The activated IKK is able to induce proteolysis of I*κ*B. The released NF-*κ*B translocates to the nucleus, binds to DNA and leads to the transcription of IAP and I*κ*B. c-IAP inhibits apoptosis by binding to caspase-3* and thus preventing DNA fragmentation. The interaction graph is depicted in Figure 4 and the notation is detailed in Table 1.

**Table 1:** The variables in the TNF*α *model

*x*_1_	TNF*α*	*x*_17_	FADD
*x*_2_	TNFR1	*x*_18_	<*x*_7_>/RIP1/FADD
*x*_3_	TNF*α*/TNFR1	*x*_19_	TRADD/TRAF2/RIP1/FADD
*x*_4_	TRADD	*x*_20_	Caspase8
*x*_5_	TNF*α*/TNFR1/TRADD	*x*_21_	TRADD/TRAF2/RIP1/FADD/Caspase8
*x*_6_	TRAF2	*x*_22_	Caspase8*
*x*_7_	TNF*α*/TNFR1/TRADD/TRAF2	*x*_23_	Caspase3
*x*_8_	RIP1	*x*_24_	Caspase8 * /Caspase3
*x*_9_	μ⟨*x*_7_⟩/RIP1	*x*_25_	Caspase3*
*x*_10_	IKK	*x*_26_	DNA *- *frag
*x*_11_	⟨*x*_7_⟩/RIP1/IKK	*x*_27_	cIAP
*x*_12_	IKK*	*x*_28_	Caspase3 * /cIAP
*x*_13_	I*κ*B/NF*κ*B	*x*_29_	DNA
*x*_14_	I*κ*B/NF*κ*B/IKK*	*x*_30_	Caspase3 * /DNA
*x*_15_	I*κ*BP	*x*_31_	I*κ*B
*x*_16_	NF*κ*B		

TNF*α *is one tumor necrosis factor which binds to the receptor TNFR1. TRADD, TRAF2 and RIP1 are adaptor proteins which may form complexes with TNF*α*. IKK, NF*κ*B, I*κ*B belongs to the NF*κ*B module while FADD, caspase8 and caspase3 are on the apoptotic pathway. c-IAP is an inhibitor of apoptosis protein.

**Figure 5:**
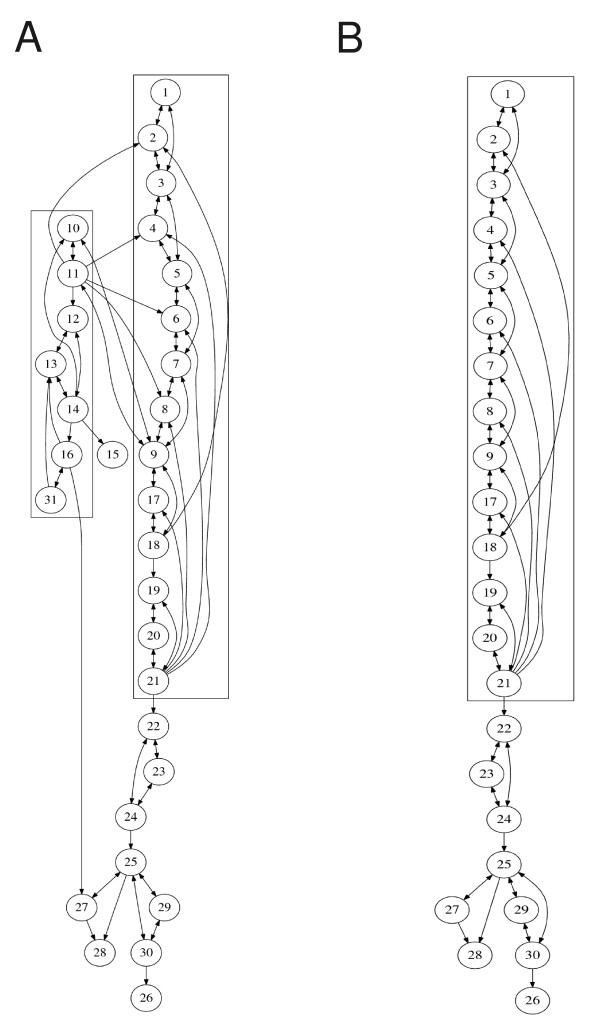
**Graph analysis of the TNF***α ***network**. (A) Feedback and forward structure through graph decomposition. (B) The minimal production unit of the TNF*α *network.

Upon application of the graph decomposition routine, we successfully unfold the underlying modular structure of the TNF-*α *network. The forward production unit is a long cascade involving many different species and reactions. The signal TNF-α (*x*_1_) is processed through the network until DNA fragmentation is induced (*x*_26_) as shown Figure 5A. The direct HVD identifies one big SCC enclosed in the two boxes in Figure 5A. Further analysis distinguishes the forward and backward edges. The whole NF*κ*B pathway is now revealed as a feedback module, which controls the level of the c-IAP (*x*_27_) and thus Caspase-3* (*x*_25_), and maintains the option for survival. It is intriguing that the NF*κ*B module is produced automatically by our decomposition procedure although it has many connections to the rest of the network. The removal of the NF*κ*B module singles out the MPU shown in Figure 5B.

**Figure 6:**
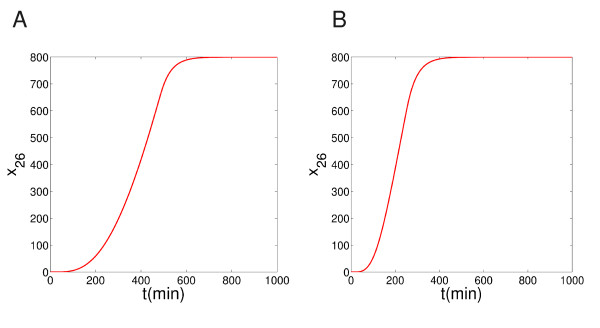
**The evolution of the DNA fragment**. The evolution of the DNA fragment (*x*_26_) (A) with and (B) without the NF*κ*B feedback module.

Figure 6 shows the level of DNA fragment (*x*_26_) with or without the presence of the NF*κ*B control module. With the feedback module, the rate of the fragmentation of DNA is low (Figure 6A), which may suggest the survival of the cell; without, the DNA cleavage is high (Figure 6B), which could indicate an apoptotic fate of the cell. So, indeed, here the NF*κ*B modules acts as a controller of the apoptotic pathway. Our decomposition technique accurately captures this information. Again, without the control module, the MPU produces over-abundantly the output signal in a relatively fast way. The long feed-forward edge from *x*_16 _to *x*_27 _may accelerate the control in this case.

### Circadian clock in Drosophila

**Figure 7:**
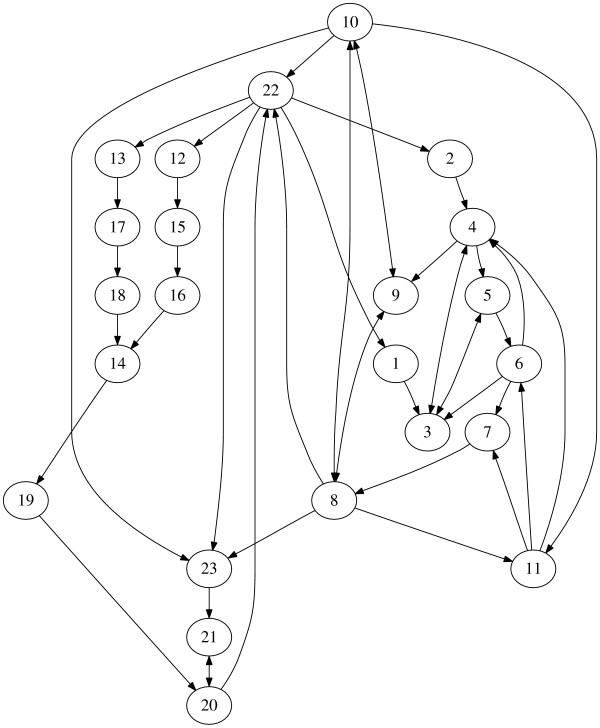
**The circadian clock model**. Network representation of the Circadian clock in *Drosophila*.

Circadian clock exists in many different organisms ranging from bacteria to human. The regulation pathway adopted from [43] and displayed in Figure 7 models the *Drosophila *circadian clock which mainly contains two interlocked loops. The notations are explained in Table 2. The TIM and PER protein in the first loop may bind to each other in the cytosol or nucleus, but they enter the nucleus separately. They down-regulate their own expression by inhibiting the transcription factor CLK-CYC. The association of TIM and PER in the cytoplasm is mediated by FBM and the dissociation is catalyzed by SM which is generated by the constitutive entering of PER into the nucleus. In the second loop, CLK-CYC activates both VRI and PDP expression. VRI represses the expression of CLK while PDP promotes it. Various forms of TIM are also influenced by the sunlight. Nevertheless, even without the coupling to sunlight, the model still produces an oscillation of period 24 hours.

**Table 2:** The variables of the circadian clock model

*x*_1_	Per*_m_*	*x*_7_	PER · P*_c_*	*x*_13_	Pdp*_m_*	*x*_19_	CLK*_c_*
*x*_2_	Tim*_m_*	*x*_8_	PER · P*_n_*	*x*_14_	Clk*_m_*	*x*_20_	CLK · CYC*_c_*
*x*_3_	PER*_c_*	*x*_9_	TIM*_n_*	*x*_15_	VRT*_c_*	*x*_21_	CLK · CYC · P*_c_*
*x*_4_	TIM*_c_*	*x*_10_	PER · TIM*_n_*	*x*_16_	VRI*_n_*	*x*_22_	CLK · CYC*_n_*
*x*_5_	PER · TIM*_c_*	*x*_11_	SM*_c_*	*x*_17_	PDP*_c_*	*x*_23_	CLK · CYC · P*_n_*
*x*_6_	PER · TIM*_f_*	*x*_12_	VRI*_m_*	*x*_18_	PDP*_n_*		

This *Drosophila *circadian clock model consists of two loops. One contains PER and TIM and the other contains PDP and VRI. They interact through CLK-CYC controlled expression. SM and FBM are two proteins assisting in the first loop.

**Figure 8:**
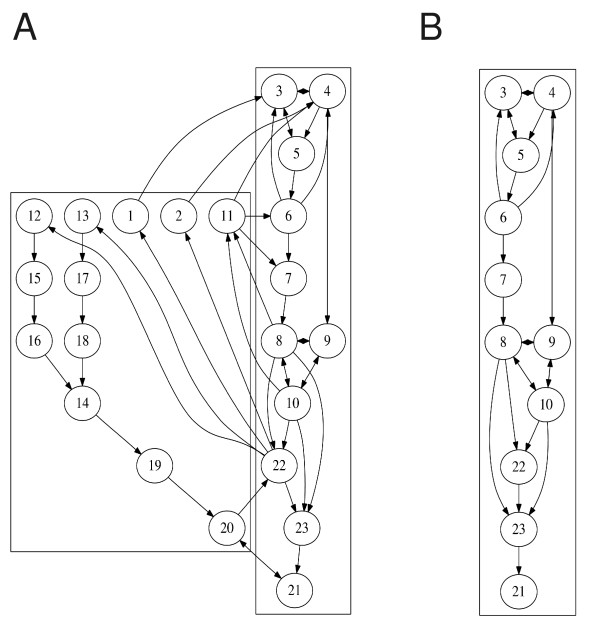
**Graph analysis of the Drosophila circadian network**. (A) Feedback and forward structure through graph decomposition. (B) The minimal production unit of the *Drosophila *circadian network.

Considering the influence of the external sunlight, we pick *x*_4 _(TIM*_c_*) as the input node while *x*_21 _(CLK·CYC·P*_c_*) is selected to be the output node since this complex controls the transcription of TIM and PER. The network graph after the decomposition analysis shown in Figure 8A clearly shows 5 feedbacks. The one through SM (*x*_11_) is the positive feedback that accelerates the dissociation of the PER·TIM complex. The other four through *x*_1_, *x*_2_, *x*_12, _*x*_13 _are the important regulators of the concentration of PER, TIM, VRI and PDP through DNA expression and protein translation. The feedbacks through *x*_12 _and *x*_13 _interact with each other and control the production of CLK (*x*_19_). The MPU is very easily obtained by removing the feedback modules and displayed in Figure 8B, which shows how the (sunlight) signal is picked up at *x*_4_, processed via PER·TIM, CLK·CYC interaction and output at *x*_21_.

**Figure 9:**
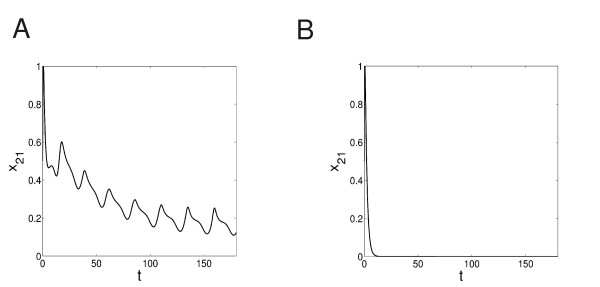
**The evolution of one important protein**. The evolution of the cytoplasmic CLK·CYC·P*_c _*(*x*_21_). (A) with all the feedbacks present and (B) of *x*_1, ⋯, 23 _without the feedback module.

With all the feedbacks, the *Drosophila *network is able to generate stable oscillations with a period of 24 hours. Indeed, employing the kinetic model in [43] and starting with a somewhat arbitrary condition, the network soon reaches an oscillatory state as shown in Figure 9. Without the feedbacks, all the state variables quickly relax to a steady state, in which concentrations are adjusted from their initial values quickly in the direction (higher or lower) corresponding to the operating point of the circadian clock. The full network follows this response in the short initial time and then feedbacks take effect to make it oscillate. So, these feedbacks are essential elements for the generation of the circadian cycles. Noticeable in Figure 9 are clearly three distinct time scales: the fastest direct response produced by the MPU, the period of the oscillation and the slowest drift to stable oscillation. The displayed feedbacks in Figure 9 are responsible for the slow adjustment of the motion and the oscillation.

## Conclusions

In this paper, we discuss some of the universal aspects of the architecture of biochemical networks that relate to their production and feedback function. We also devise an automatic procedure for identifying the key functional modules of that architecture by applying graph theoretic methods and invoking additional dynamic information. The key ingredients of the architecture are revealed by identifying the forward production unit and the feedback controller. We successfully applied the HVD and the feedback loop searching and selection algorithm and obtained this anatomy in the NF*κ*B regulatory, the *E. coli *chemotaxis network, the TNF-*α *pathway and the circadian network. In the Additional File 1 we show that similar structures exist in a number of other cell regulatory networks.

The dissection of large networks into functional modules greatly facilitates their analysis. The functional modules can be studied individually with well-designed boundary conditions. The properties of the whole network are deducible by piecing together the modules in an ordered way. Henceforth, our strategy of analysis is characterized by a decomposition and recombination procedure. Current technique can be further extended to the analysis of hierarchical structures at different scales with disparate internal dynamics. In the top-down direction, the network may be broken into functional modules at different scales by the above decomposition technique. From bottom up after the property of each module is conveniently explored, a hierarchy of modules of increasing size may be built until the whole network is covered. From biological evolution point of view, it is likely that this nested structure stems from a simple core and is later wrapped with complex regulation mechanisms during evolution. So, our theory reveals the stable, potentially generic feature of a biochemical network, which can be used to explore either the intricacy in a single structure or interdependencies of a series of systems.

The detection of modular structures provides additional insight into how a regulatory network works and thus gives clear indication of key protein species and key reactions in a cascade, which finds wide applications in the drug design and synthetic biology [44]. The identification of the dominating skeleton subnetwork such as the MPU and key feedbacks in a regulatory pathway also simplifies the determination of reaction rates of *in vivo *biochemical reaction since the distracting unimportant reaction components have been removed from the skeleton structure [45,46]. In all, the production and feedback dichotomy of biological networks shapes cellular signaling [22] and the current graph decomposition technique provides a convenient handle to uncover this important aspect of their architecture.

## Methods

### Identification of forward and feedback edges

As mentioned previously, here, we present an algorithm to identify the forward and feedback edges with given polarity, by searching and ordering important topological invariants - cycles. First, a cycle search procedure is discussed which produces all the cycle generators for a strongly connected component. Then a selection procedure is discussed which generates a partial order of the vertices and enables the detection of feedbacks in a straightforward way. Before proceeding directly to the algorithm part, we state a principle which will be used in our selection procedure.

#### Principle of minimum feedbacks

Very often, in complex systems, multi-step processes are carried out in a well-ordered sequential way with a small number of feedback controllers modulating the behavior of the system. The cascade structure with minimal number of feedback controls yields balance between robustness and evolvability. It also has the advantage of maximizing operation efficiency and minimizing energy cost. As an analogue, we propose that in order to make optimal use of resources and at the same time maintain necessary stability cells employ a minimum feedback principle: the number of feedback edges should be minimal in a cell regulation network. It seems evolutionarily advantageous to allocate only necessary resources to feedback control. As always happens in biology, there may exist other requirements which weaken this principle. Here, we just stick to this principle which produces reasonable results for all the examples we are looking into so far.

How to find a minimum set of feedback edges is an NP-hard problem in graph theory but there exist approximate algorithms which could do the job relatively fast [47]. It is conceivable that the solution might not be unique. However, extra constraints may help remove some non-uniqueness. From a control theory point of view, the signal transduction network consists of two major components, the information forwarding part and the feedback controller. The forwarding part receives external signal at one end, passing and processing it along different paths, and producing an output at the other end. So, the associated information flow defines a direction on the network. The feedback component modulates the flow by sending downstream signals back to upstream nodes. The identification of these two components is essential for understanding the function of different parts of a network. The problem of searching for the minimal set of feedback arcs has to be consistent with the polarity determined by the information flow. Accordingly, we may restate the problem in an equivalent way: find an ordering of the vertices with the given polarity determined by input and output vertices, such that the number of feedback edges is minimized.

#### Cycle search

**Figure 10:**
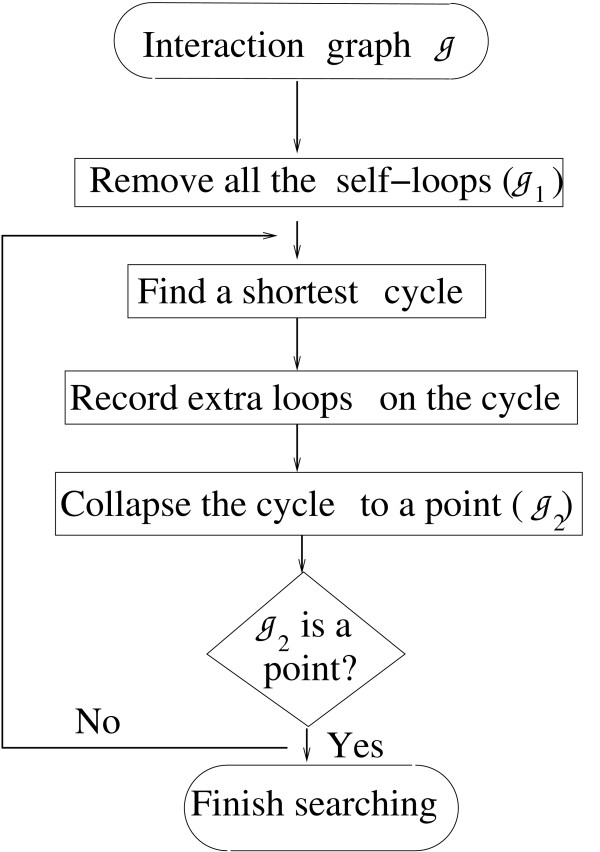
**The flow chart for cycle search**. The procedure displayed here is applicable to the strongly connected component (SCC) of a network. The result is a set of cycle generators.

For a finite graph, there exist sets of linearly independent cycles, the algebraic combination of which is able to produce all cycles in the graph. Such a set is called the cycle generator set, denoted by *C*gen in our paper. It is not unique, but the number of elements in *C*gen is fixed for different sets and determined by the graph itself. Therefore, for an SCC, all the edges lie in the generator set *C*_gen_. In the following, we introduce a collapsing scheme to find one *C*_gen _of a general graph G = {*a_i _*: *i *= 1, 2, ⋯, *m*|*v_i _*: *i *= 1, 2, ⋯, *n*}, where *a_i_*'s represent vertices of G, and *v_i _*= (*j*, *k*) = [*a_j_*, *a_k_*] represents an edge from the vertex *a_j _*to the vertex *a_k_*. A chain of edges, denoted by $\left[a_{i_{1}},a_{i_{2}}],\cdot \cdot \cdot ,a_{i_{k}}\right]$, represent a path from $a_{i_{1}}$ to $a_{i_{2}}$ to ... and finally to $a_{i_{k}}$. We use ${\left[a_{i_{1}},a_{i_{2}}\right]}^{-},\cdots ,a_{i_{k}}]$ to represent a cycle of *k*-nodes. The adjacency matrix of G is denoted with *A*. The main idea is to identify shortest cycles and then simplify the graph in an iterative way and a flow graph of the procedure is shown in Figure 10 which is explained below in detail.

(1) Record all the self-loops of G which are encoded by the nonzero diagonal elements of *A*. After removing the corresponding edges from G, we obtain a new graph $G_{1}$ and a new adjacency matrix *A*_1_.

(2) Search and record a shortest cycle $l_{1}=\bar{\left[a_{i_{1}},...,a_{i_{k}}\right]}$ of *A*_1 _for some *k *> 1 by looking for the nonzero diagonal elements of the *m*-th powers of *A*.

(3) The induced subgraph $H_{1}$ with the vertex set {*a*_1_,..., *a_m_*} and their connections has an adjacency matrix *B*_1 _which is a submatrix of *A*_1_. Each nonzero element (*i*_*p*_, *i*_*p*+1_) of *B*_1 _can be made to a cycle by connecting $a_{i_{p+1}}$ back to $a_{i_{p}}$ with part of the cycle *l*_1_, *e.g*., by the chain of edges $\left[a_{i_{p+1}},a_{i_{p+2}},...,a_{i_{p}}\right]$. Initially, this step is not necessary since besides *l*_1 _there is no extra edge in $H_{1}$. However, after the collapse in step 4, there may appear multi-edges between some pair of nodes. For each of those in $H_{1}$ but not in *l*_1_, we can identify and record a new cycle.

(4) Collapse all the edges and vertices in the subgraph $H_{1}$ into one point *P*_1_, and we obtain the updated graph $G_{2}$ for which a new adjacency matrix *A*_2 _is written down. If $G_{2}$ only contains *P*_1_, the iteration is terminated. Otherwise, we go back to step 2 and repeat the procedure with the new graph $G_{2}$ and the new adjacency matrix *A*_2_. Note that $G_{2}$ may not be a simple graph: there could be more than one edge between some pair of vertices. This is the origin of extra edges on a shortest cycle in step 3.

It is easy to show that each cycle of $G_{1}$ corresponds to a unique cycle either in $H_{1}$ or in $G_{2}$. Vice versa, each cycle *l *in $G_{2}$ can be identified with a unique cycle in $G_{1}$: if the cycle *l *runs through *P*_1_, then its incidence vertex and exit vertex in $H_{1}$ can be connected by a unique path embedded in the cycle *l*_1 _and thus a unique cycle in $G_{1}$ is produced by concatenating this path to the edges contained in *l*; if the cycle *l *stands apart from *P*_1_, it directly corresponds to one cycle in $G_{1}$. So, after the search is done, finally, we can trace backward all the cycles we have found so far in the original graph G except the self-loops. In the algorithm just described, not all cycles but a set of linearly independent cycles are recorded, which by definition constitutes a cycle generator set *C*gen. The generators derived from the above algorithm are prime in the sense that any proper subset of a generator is not a cycle. Note that the set *C*gen may not be unique since the selected cycle in step 2 might not be unique. What consequences this non-uniqueness brings about is an interesting problem that deserves further investigation. However, the important point here is that all the feedback edges appear at least once in *C*gen.

For the NF-*κ*B gene regulatory network, we apply the cycle-searching technique and find that the total number of cycle generators are 33 with 15 1-cycles and 8 2-cycles. 10 cycle generators have length greater than 2.

#### Selection procedure

**Figure 11:**
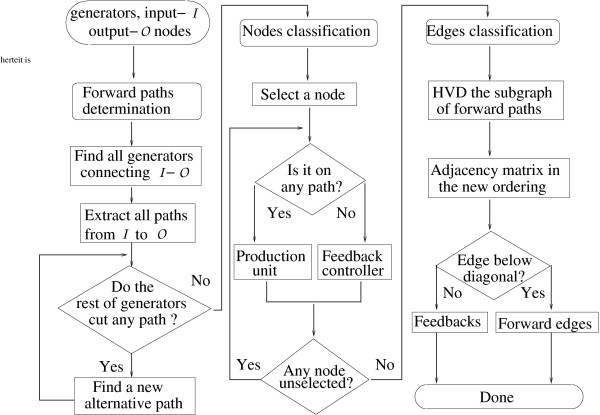
**The flow chart for cycle selection**. Started with the cycles already determined and given polarity, the procedure here identifies the forward part and the feedback part of the network.

For a graph with a tree structure, it is always possible to find an order of the vertices, such that only forward edges show up. For instance, the HVD of the graph could generate such an ordering. With cycles present, at least one feedback exists no matter how the vertices are ordered. According the principle of minimal feedbacks, we want to find an ordering under which the number of feedbacks is minimized. That is to say, we set out to extract a minimal set of the edges, the removal of which leads to a cycle-free network. It is not uncommon that by removing one edge quite a few cycles get destroyed. In order to determine the forward and feedback edges in an SCC based on the cycles found in the previous section, we utilize the polarity of the network that determines the flow direction. In a graph possessing polarity, for the input vertex, every out-edge is regarded as a forward edge and every in-edge a feedback. The opposite is true for the output vertex. Note that 1-cycles (self-loops) and 2-cycles are special and need to be treated differently. 1-cycles always attach to individual vertices and are not regarded as feedback loops. 2-cycles are bidirectional edges that are most likely representing the forward and the backward reactions since many of biochemical reactions are reversible. These 2-cycles are important for keeping chemical balance but not usually regarded as feedbacks from a signal transduction point of view. Each 2-cycle contributes exactly one feedback edge and one forward edge, irrespective of the ordering of the vertices. Therefore, they have no impact on the vertex ordering regarding the search of minimal set of feedbacks. Hence, in the selection procedure, we only consider cycles of length greater than or equal to 3 and we call them "long cycles". We take the middle layer of the NF*κ*B network from the HVD result as an example. Here, the input point is *x*_2 _as it receives signals from *x*_1 _and the output point is *x*_7 _as it sends signals to *x*_15_. The goal is to find all the forward paths that go from *x*_2 _to *x*_7 _and all the feedback loops. The algorithm is depicted as a flow chart in Figure 11 and a detailed explanation is given below.

(1) With the long cycles and the polarity determined, we first look for cycles connecting *x*_2 _and *x*_7 _and thus extract a set of forward paths that go from *x*_2 _to *x*_7_.

(2) From the remaining long cycles, we search for the ones intersecting an extracted path at two nodes. If more than two intersections are found, we choose the two intersections that are most separated. This choice is to put as many edges as possible to the forward direction and thus to minimize the feedback ones. Using the edges on the cycle as a replacement of the edges in the path that connect the two intersections, an alternative path from *x*_2 _to *x*_7 _is constructed.

(3) We repeat the search until no more alternative paths can be generated from the available long cycles.

(4) Now, it is possible to construct a subgraph F expanded by the vertices and the edges contained in these forward paths. A node in F belongs to the production unit and to the feedback controller otherwise. For the middle layer of the NF*κ*B network, the vertex set in F has been computed as *V_f _*= {*x*_2 _, *x*_5 _, *x*_6 _, *x*_7 _, *x*_10 _, *x*_11 _, *x*_13_} which sit in the forward production unit and are displayed inside the rectangle in Figure 2A. The complementary vertex set consists of *V_b _*= {*x*_4 _, *x*_8 _, *x*_9 _, *x*_12 _, *x*_14_} which should be included in the feedback controller.

(5) The HVD is applied to F to partially order its vertices and edges. We rearrange the order of the vertices in F according to the partial order. In the new order, an adjacency matrix only has subdiagonal nonzero entries, which represent forward edges. If we restore all edges in the original graph that connect nodes in F, the adjacency matrix may have superdiagonal entries, which are considered as feedback edges. For the NF*κ*B network, the collection of the feedback and the forward edges are clearly seen in the rectangle in Figure 2A. For a complex feedback controller, if needs arise, we may carry out further decomposition with our cycle search and selection algorithm. For the NF*κ*B network, it is not necessary since the feedback controllers are simple line graphs.

## Authors' contributions

IM conceived the dichotomy structure of cell regulation network and emphasized the importance of the feedback loops in understanding network function. YL designed the decomposition scheme based on the cycle search and selection and did the analysis on various networks. Both authors read and approved the final manuscript.

## Supplementary Material

Additional file 1 **Examples of several other cell regulation networks**. Click here for file
