# Supplementary material for: On the architecture of cell regulation networks
Source: BMC Syst Biol. 2011 Mar 2;5:37. doi: 10.1186/1752-0509-5-37 (PMC3060115; doi:10.1186/1752-0509-5-37)
Supplement: Additional file 1 — Examples of several other cell regulation networks. [file 1752-0509-5-37-S1.PDF]

More applications on cell regulation networks

Yueheng Lan and Igor Mezić

September 10, 2010

In the first section, we give a detailed description of the NFκB network in the main text. In the rest sections, we implement the decomposition scheme on several typical cell regulation networks. It turns out that the MPU-controller dichotomy structure seems to be universal and the decomposition based on the cycles is able to capture the modular information embedded in a complex network.

1 Chemical kinetic equations of the NFκB network

Deterministically, the dynamics of the NFκB network is described by a set of chemical kinetics equations:

$$\begin{aligned}
\dot{x_1} &= k_{prod} - k_{deg}x_1 - k_1ux_1 \\
\dot{x_2} &= k_1ux_1 - k_3x_2 - k_2ux_2x_8 - k_{deg}x_2 \\
&\quad -a_2x_2x_{10} + t_1x_4 - a_3x_2x_{13} + t_2x_5 \\
\dot{x_3} &= k_3x_2 + k_2ux_2x_8 \\
\dot{x_4} &= a_2x_2x_{10} - t_1x_4 \\
\dot{x_5} &= a_3x_2x_{13} - t_2x_5 \\
\dot{x_6} &= c_{6a}x_{13} - a_1x_6x_{10} + t_2x_5 - i_1x_6 \\
\dot{x_7} &= i_1k_vx_6 - a_1x_7x_{11} \\
\dot{x_8} &= c_4x_9 - c_5x_8 \\
\dot{x_9} &= c_2 + c_1x_7 - c_3x_9 \\
\dot{x_{10}} &= -a_2x_2x_{10} - a_1x_6x_{10} + c_{4a}x_{12} - c_{5a}x_{10} \\
&\quad -i_{1a}x_{10} + e_{1a}x_{11} \\
\dot{x_{11}} &= -a_1x_7x_{11} + i_{1a}k_vx_{10} - e_{1a}k_vx_{11} \\
\dot{x_{12}} &= c_{2a} + c_{1a}x_7 - c_{3a}x_{12} \\
\dot{x_{13}} &= a_1x_6x_{10} - c_{6a}x_{13} - a_3x_2x_{13} + e_{2a}x_{14} \\
\dot{x_{14}} &= a_1x_7x_{11} - e_{2a}k_vx_{14} \\
\dot{x_{15}} &= c_{2c} + c_{1c}x_7 - c_{3c}x_{15}\,, \qquad \text{(S1)}
\end{aligned}$$

where the notation is adopted from the reference [1] and marked in Fig. 1A.Protein IKK has three different forms, the neutral form IKK$_n$ (x_1), the activated form IKK$_a$ (x_2) and the deactivated form IKK$_i$ (x_3). IKK$_n$ is passive but can be activated into IKK$_a$

by external cues like TNF or IL-1. $u \in \{0, 1\}$ is a switch variable which is equal to one when the external cue is present but equal to zero otherwise. IKK_a is deactivated by A20 (x_8) into IKK_i (x_3) which is different from IKK_n and cannot be activated. IKK_a is able to bind with IκBα (x_{10}) to form the protein complex IκBα-IKK_a (x_4) or with the complex IκBα-NFκB (x_{13}) to form a tri-molecular complex IKK_a-IκBα-NFκB (x_5). Once IKK_a binds with IκBα, in either the bimolecular or the trimolecular form, it phosphorylates and initiates proteolysis of IκBα so that NFκB (x_6) is released from the complex and restores its enzymatic capability, entering the nucleus and being denoted by NF$\kappa$$B_n$ (x_7). When NF$\kappa$$B_n$ binds to the relevant promoter regions in the DNA, a variety of transcriptions are initiated. The transcripts (x_9) for A20 and the transcripts (x_{12}) for IκBα move to cytoplasm and start synthesis of the corresponding proteins. Other interesting transcripts (x_{15}) for certain signaling proteins may be generated as well. The newly synthesized protein IκBα will enter the nucleus, wherein denoted by IκBα_n (x_{11}), and bind with NF$\kappa$$B_n$ to form the complex IκBα-NF$\kappa$$B_n$ (x_{14}), leaving the nucleus for the cytoplasm. Thus, the transcription is temporarily terminated by the association with protein IκBα. The switch protein IKK_a itself is also constantly deactivated by A20. In the mean time, almost all the proteins and transcripts decay spontaneously, and the bindings and unbindings all have constitutive reaction rates which are much smaller than the corresponding enzymatic reaction rates.

2 B. Subtilis chemotaxis network

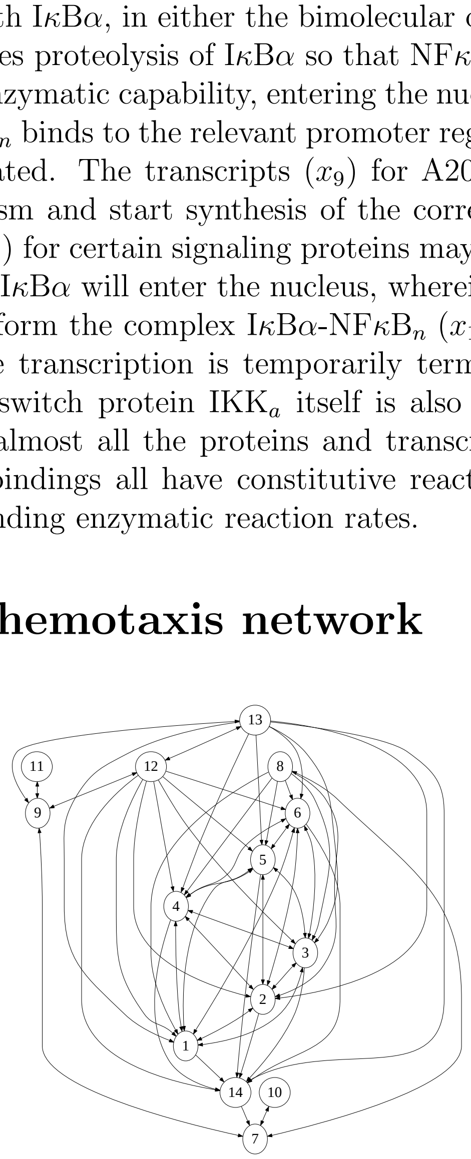

Figure S1: Network representation of the chemotaxis model of *B. Subtilis*.

The chemotaxis signaling network of *B. subtilis* retains many features of that of *E. coli* but also has varied considerably. The model is adapted from [2]. See Fig. S1 for the interaction graph and the notation is explained in Table S1. Here, in a similar way, the receptor (T) adopts different configurations (active, inactive, weakly active, weakly inactive) according to its methylation states and external ligand concentration. However, the activation and deactivation of the whole receptor complex (R) also depends on the binding of $CheY_p$. The active receptor complex can activate CheA which in turn activates CheY. The phosphorylated CheY binds to the flagellar protein and enhances straight runs of the bacterium. It is also assumed that $CheY_p$ deactivates CheA. $CheA_p$ also activates CheB which adjusts the methylation state of the receptor. A new

Table S1: T_{ij} denotes six different methylation states of the receptor dimer where the index i and j mark the methelation states of residue 630 and 637, respectively. CheABVY denote different kinases in chemotaxis signaling. RY_p, R_Y denote receptors with and with no ChemY_p binding, while R^A denotes activated receptors

x_1	T_{20}	x_6	T_{11}	x_{11}	Motor − CheY_p
x_2	T_{10}	x_7	CheA_p	x_{12}	R_Y
x_3	T_{00}	x_8	CheB_p	x_{13}	RY_p
x_4	T_{01}	x_9	CheY_p	x_{14}	R^A
x_5	T_{02}	x_{10}	CheV_p		

inhibitor CheV exists in *B. subtilis* network which disrupts the receptor complex when unphosphorylated but can be deactivated by CheA_p phosphorylation.

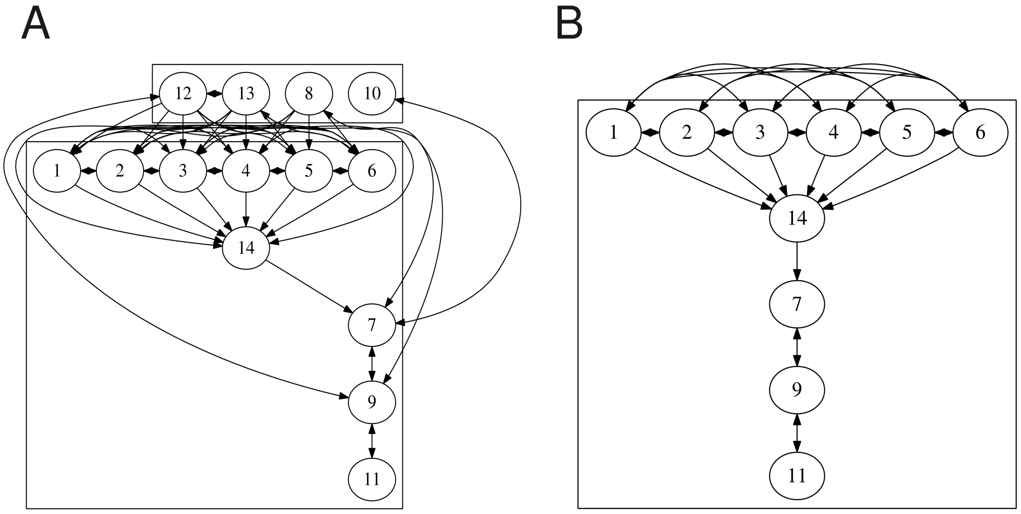

Figure S2: Graph decomposition and the MPU of the *B. Subtilis* chemotaxis network. (A) The feedback and forward structure through graph decomposition. (B) The MPU of the chemotaxis network.

Through the graph decomposition discussed previously, three main feedbacks are unambiguously detected and displayed in Fig. S2A for the *B. Subtilis* chemotaxis network. One is through x_8 (CheB_p) which is activated by x_7 (CheA_p) and changes the methylation state of the receptor ($x_{1,\cdots,6}$). The feedback through x_{10} (CheV_p) is a controller of the receptor complex that does not exist in the *E. Coli* chmotaxis network while the feedback through x_{12} and x_{13} relays the action of CheY_p (x_9) to regulate the activation and inactivation of the receptor complex. The first six variables $x_{1,\cdots,6}$ representing different methylation states are interconnected, making up a complete subgraph and describing their interdependence. All of them affect the activation of the whole receptor complex (x_{14}).

We checked the dynamics by using the kinetic equations and parameter values in [2]. Fig. S3 shows the change of x_9 (CheY_p) in the time interval $[0\,,1500]$, starting with $x_9 = 0$, $10\mu M$ attractant added at $t = 500$ and removed at $t = 1000$. When the full network is put to work, the adaptation is clearly exhibited in Fig. S3A by the elevation of x_9 during $[500\,,1000]$ in the presence of the chemo-attractant. When the feedback

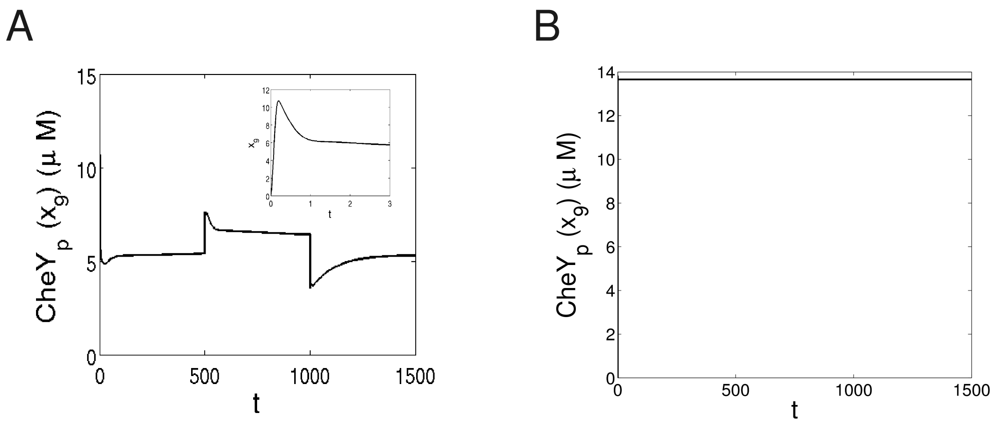

Figure S3: The concentration change of CheY$_p$ (x_9) of the chemotaxis network of *B. Subtilis*. (A) with and (B) without feedbacks. External attractant of $10\mu M$ is added at $t = 500$ and removed at $t = 1000$. The insets in (A) and (B) display the fast initial transient.

loops are cut (*e.g.* the system in Fig. S2B is simulated), we get in Fig. S3B a stable value of x_9, which does not change with the attractant concentration. So the subgraph shown in Fig. S2B is an MPU, which only provides the basic supply of CheY$_p$, devoid of capability of adaptation. Also noticeable is the extremely fast response produced by the MPU as displayed in the insets of Fig. S2A, B and the slow adaptation process controlled by the feedbacks.

Comparison of Fig. 3 and S2 reveals the similarity and difference between the chemotaxis pathways of *E. coli* and *B. subtilis*. The forward MPU parts of both pathways are almost identical. Nevertheless, the feedback regulator in *B. subtilis* has extra loops and layers which may provide more subtle control [2].

Table S2: R denotes the EGF binding receptor. PLC and PLP denotes the enzyme PLC_γ and its phosphorylated form, respectively. G and S stand for Grb and SOS. ShP is the phosphorylated form of Shc

x_1	EGF	x_7	$EGFR_p$–PLP	x_{13}	$EGFR_p$–ShP – G – S	x_{19}	PLC – I
x_2	R	x_8	$EGFR_p$–G	x_{14}	G – S	x_{20}	G
x_3	EGF – R	x_9	$EGFR_p$–G – S	x_{15}	ShP	x_{21}	Shc
x_4	EGF – R_2	x_{10}	$EGFR_p$–Shc	x_{16}	ShP – G	x_{22}	S
x_5	EGF – R_{2p}	x_{11}	$EGFR_p$–ShP	x_{17}	PLC	x_{23}	Shc – G – S
x_6	$EGFR_p$–PLC	x_{12}	$EGFR_p$–ShP – G	x_{18}	PLP		

3 The EGF receptor signaling

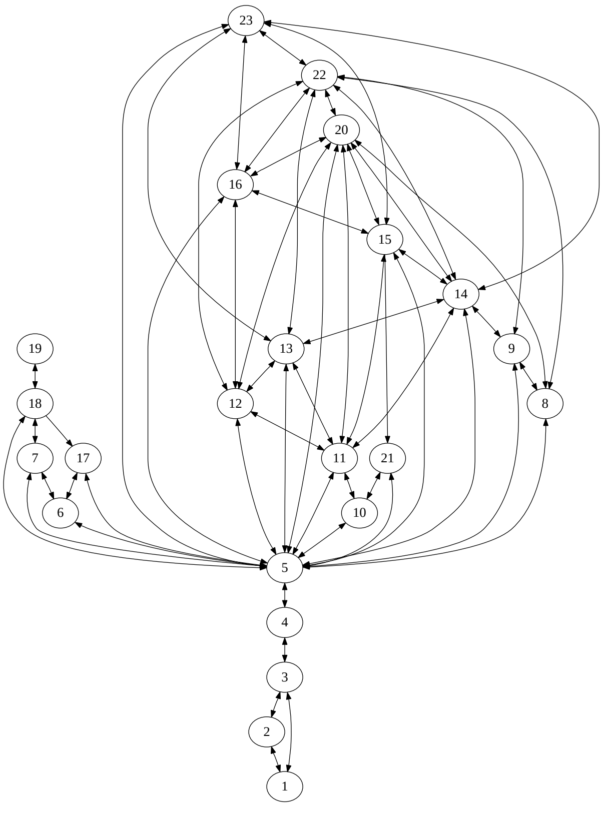

Figure S4: Network representation of the epidermal growth factor receptor model.

The epidermal growth factor receptor (EGFR) belongs to the family of protein-tyrosine kinase receptors, which regulate cell growth, survival, proliferation and differentiation [5, 6]. We consider a simplified version of the EGFR signal transduction network adopted from [7]. The interaction graph is depicted in Fig. S4 and notations are explained in Table S2. Upon binding of EGF (x_1) to its receptor R (x_2), the occupied receptors EGFR (x_3) will form dimers $EGFR_2$ (x_4) and get phosphorylated $EGFR_{2p}$ (x_5), which triggers the binding of the cytoplasmic proteins, such as Grb2 (x_{20}), Shc(x_{21}) and PLCγ (x_{22}), to the receptor. PLCγ binds to the receptor to form a complex $EGFR_p$-PLC (x_6) and gets phosphorylated $EGFR_p$-PLP (x_7). The activated PLCγ, *i.e.* PLP (x_{18}) then dissociates with the receptor and initiates many subsequent reactions, like binding to cytoskeletal structures PLC-I (x_{19}). The complex $EGFR_p$-G (x_8) of the adaptor pro-

tein Grb2 and the receptor may recruit the GDP-GTP exchange factor SOS (x_{22}) and put it in the vicinity of Ras. The binding of Grbs to the receptor could also be mediated by Shc, which makes a complex EGFR$_p$-Shc (x_{10}) first and is consequently phosphorylated into EGFR$_p$-ShP (x_{11}). The binary complex is able to bind Grb2 to form a ternary complex EGFR$_p$-ShP-G (x_{12}) and then binds to SOS to produce a quadruple EGFR$_p$-ShP-G-S (x_{13}), which approaches and affects the Ras activity like in the complex EGFR$_p$-G-S (x_9). All the complexes dissociate at some rates.

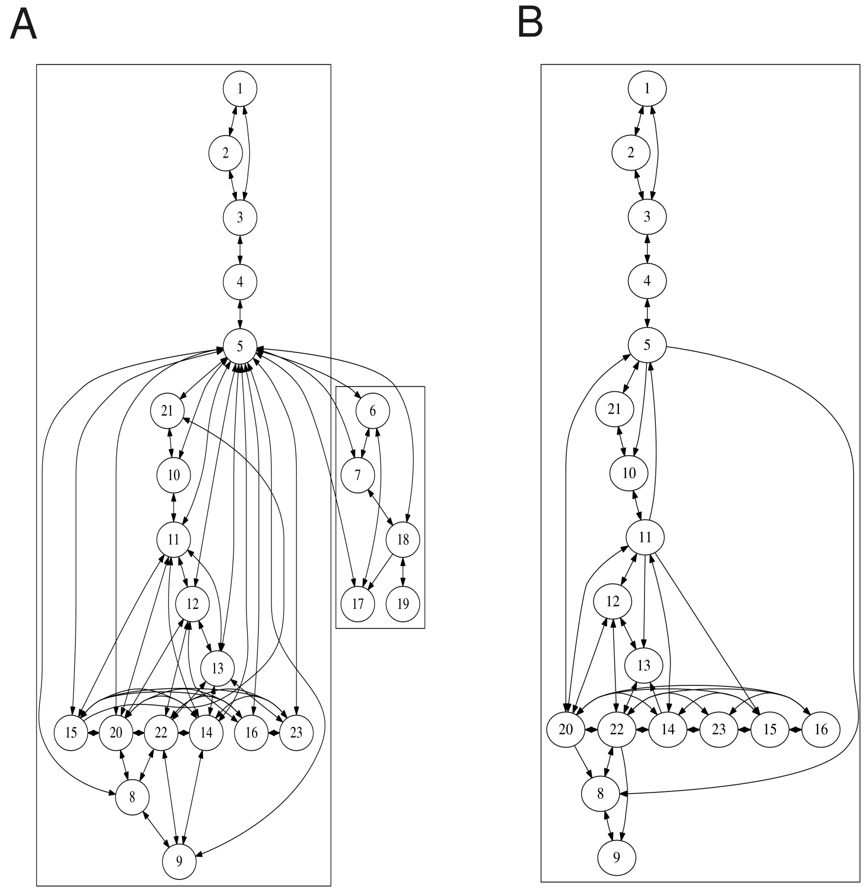

Figure S5: Graph analysis of the EGFR network. (A) Feedback and forward structure through graph decomposition. (B) The minimal production unit of the EGFR network.

In this example, almost all reactions are reversible so the whole network is strongly connected and hence usual simple decomposition schemes do not perform well. Our scheme, however, is still able to generate the biologically meaningful topology if the input and output vertices are appropriately selected, as shown in the decomposed graph in Fig. S5A. The binding, dimerization and phosphorylation of the EGFR leads to the production of EGF-R$_{2p}$ (x_5) which becomes the hub of all the signaling processes. The reactions involving PLC (x_{17}) are selected by the procedure to make one separate module in the small box on the right of Fig. S5A. This is biologically plausible since they are not directly related to the production of EGFR$_{2p}$-G-S (x_9). Displayed in the graph are mainly three routes to the target output x_9. The longest one is through $x_{21} \to x_{10} \to x_{11} \to x_{12} \to x_{13}$, another is through x_8 and the shortest one is a direct connection from x_5 to x_9. But the shortest one is related to the reverse reaction of the dissociation of the EGFR$_{2p}$-G-S complex and should not be treated as a signaling

process. It is removed in the MPU graph shown in Fig. S5B. So, there are two major pathways as explained before: the short one passing x_8 is through direct binding of Grb and the other long one is mediated by the binding and activation of Shc. The MPU shown only contains the short course.

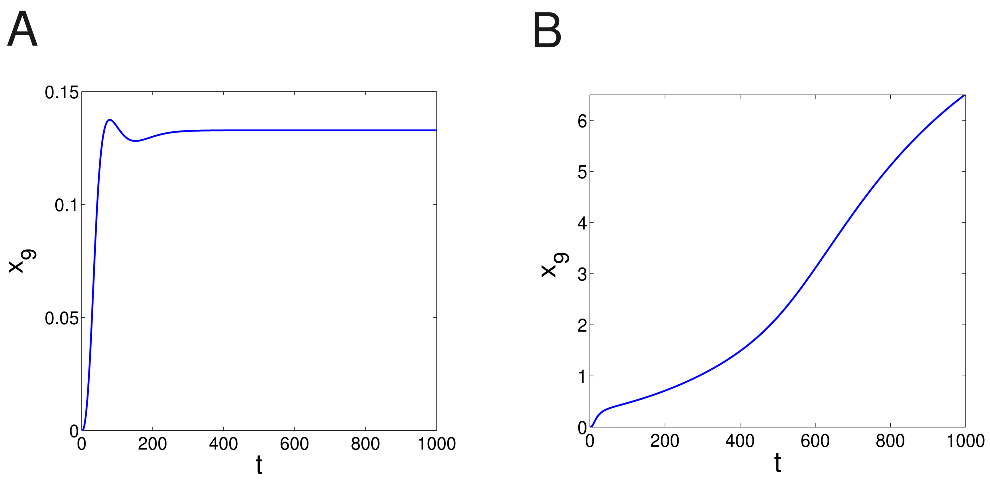

Figure S6: The evolution diagram of the EGFR$_p$-G-S (x_9). (A) with all the feedbacks present and (B) with MPU.

The time evolution of the output plotted in Fig. S6 shows that with the feedback control (Fig. S6A), the signaling arrived at the desired value $x_9 = 0.13$ in a short time and stay there indefinitely long. Working only with MPU (Fig. S6B), however, the signal quickly rises to a large steady value $x_9 = 4.5$ and might change the behavior of the downstream reaction as a consequence. As before, the fast production unit responds in a rapid and uncontrolled manner while the extra controllers may pin down the output to the desired value at a large time scale.

4 G-protein coupled receptor model

Here, the model is taken from [8], for the G protein-coupled receptor (GPCR) signaling modules in macrophage immune cells. The GPCR system responds to multiple external cues, such as: light, hormones, odorants, neurotransmitters, amino acids. The system is an important drug target and is one of the most common signaling channels in a cell. Here, a simplified model is used to study the effects of two signaling molecules (C5a and UDP) on the second messenger calcium Ca^{2+}.

The interaction graph of the model network is displayed in Fig. S7 and the notation is explained in Table S3. The main biochemical process could be explained as follows. Upon C5a and UDP binding to specific cell surface receptors, the G-protein heterotrimer dissociates to free Gα-GTP and G$\beta\gamma$, both being able to bind isoforms of PLCβ and catalyze the synthesis of IP3 and DAG from PIP2. IP3 binds to certain ion channels on the membrane of endoplasmic reticulum to induce the release of Ca^{2+} into the cytosol. DAG and Ca^{2+} bind to and activate PKC which then phosphorylates and inactivates PLCβ. GRK is localized at the membrane by G$\beta\gamma$ once it is phosphorylated by PKC. GRK phosphorylates and inactivates C5a receptor (C5aR). There is also Ca^{2+} flow from other buffers in the cell or from extracellular environment.

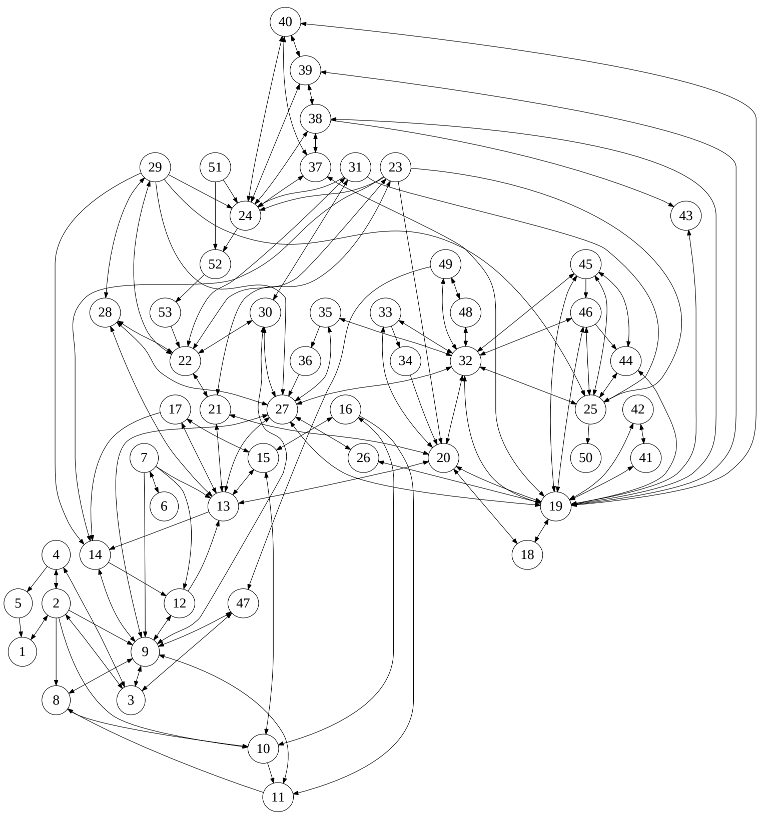

Figure S7: Network representation of the G-protein coupled receptor model.

Upon application of our decomposition technique to the above network, a structured graph is obtained and displayed in Fig. S8. The network consists of four parts:

- The source vertex set $\{x_6\,, x_7\,, x_{51}\}$.
- The sink vertex set $\{x_{50}\}$.
- The feedback vertex set $\{x_5\,, x_{34}\,, x_{41}\,, x_{42}\,, x_{48}\,, x_{49}\,, x_{52}\,, x_{53}\}$.
- The major strongly connected processing unit, the rest of the vertices

The cycle search and selection program decomposed the major processing part into horizontal layers, put in an order which relays the signals input from vertex x_1, the cell surface receptor, and terminates at vertex x_{19}, the calcium ion. The major feedbacks recognized in the literature [8] are precisely identified by our automatic procedure: the route through $x_{52}\,, x_{53}$ marks the feedback of phosphorylation of IP3 back to PIP2; the one through $x_{48}\,, x_{49}$ marks the feedback of PKC on GRK2; the one through x_{34} marks the action of calcium on isforms of PLCβ; the one through $x_{41}\,, x_{42}$ marks the exchange of calcium with other calcium buffers. Our program also identified many more feedbacks which are more local and visible in the boxed forward processing unit. The MPU is shown in Fig. S8B where we have removed the feedback box and some of the inessential feedbacks for production in the forward processing unit. Even without removal of this, the fast response is present.

Shown in Fig. S9A, is the change of Ca^{2+} over time when a concentration $250nM$ of C5a is added at $t = 0$. A fast initial growth of x_{19} and a slow relaxation to a steady

Table S3: The notations for the G-protein coupled receptor model. Buf represents other calcium buffers in the cell

x_1	C5aR	x_{21}	$\mathrm{PLC}\beta4 \cdot \mathrm{Ca}^{2+} \cdot \mathrm{G}\alpha_q\mathrm{GTP}$	x_{41}	Buf
x_2	C5aC	x_{22}	PIP_2	x_{42}	$\mathrm{Ca}^{2+} \cdot \mathrm{Buf}$
x_3	$\mathrm{GRK}_p \cdot \mathrm{G}\beta\gamma$	x_{23}	$\mathrm{PLC}\beta_4 \cdot \mathrm{Ca}^{2+} \cdot \mathrm{G}\alpha_q\mathrm{GTP} \cdot \mathrm{PIP}_2$	x_{43}	Ca^{2+}_{ER}
x_4	$\mathrm{GRK}_p \cdot \mathrm{G}\beta\gamma \cdot C5aC$	x_{24}	IP3	x_{44}	PKC
x_5	$\mathrm{C5aC}_p$	x_{25}	DAG	x_{45}	$\mathrm{PKC} \cdot \mathrm{DAG}$
x_6	P2YR	x_{26}	$\mathrm{PLC}\beta3$	x_{46}	$\mathrm{PKC} \cdot \mathrm{Ca}^{2+}$
x_7	UDPC	x_{27}	$\mathrm{PLC}\beta3 \cdot \mathrm{Ca}^{2+}$	x_{47}	GRK_p
x_8	$\mathrm{G}\beta\gamma \cdot \mathrm{G}\alpha_i\mathrm{GDP}$	x_{28}	$\mathrm{PLC}\beta3 \cdot \mathrm{Ca}^{2+} \cdot \mathrm{G}\alpha_q\mathrm{GTP}$	x_{48}	GRK
x_9	$\mathrm{G}\beta\gamma$	x_{29}	$\mathrm{PLC}\beta_3 \cdot \mathrm{Ca}^{2+} \cdot \mathrm{G}\alpha_q\mathrm{GTP} \cdot \mathrm{PIP}_2$	x_{49}	$\mathrm{PKC} \cdot \mathrm{DAG} \cdot \mathrm{Ca}^{2+} \cdot \mathrm{GRK}$
x_{10}	$\mathrm{G}\alpha_i\mathrm{GTP}$	x_{30}	$\mathrm{PLC}\beta3 \cdot \mathrm{Ca}^{2+} \cdot \mathrm{G}\beta\gamma$	x_{50}	DAG_d
x_{11}	$\mathrm{G}\alpha_i\mathrm{GDP}$	x_{31}	$\mathrm{PLC}\beta3 \cdot \mathrm{Ca}^{2+} \cdot \mathrm{G}\beta\gamma \cdot \mathrm{PIP2}$	x_{51}	$\mathrm{IP3}K_a$
x_{12}	$\mathrm{G}\beta\gamma \cdot \mathrm{G}\alpha_q\mathrm{GDP}$	x_{32}	$\mathrm{PKC} \cdot \mathrm{DAG} \cdot \mathrm{Ca}^{2+}$	x_{52}	IP4
x_{13}	$\mathrm{G}\alpha_q\mathrm{GTP}$	x_{33}	$\mathrm{PKC} \cdot \mathrm{DAG} \cdot \mathrm{Ca}^{2+} \cdot \mathrm{PLC}\beta4 \cdot \mathrm{Ca}^{2+}$	x_{53}	IP5
x_{14}	$\mathrm{G}\alpha_q\mathrm{GDP}$	x_{34}	$\mathrm{PLC}\beta_4 \cdot \mathrm{Ca}^{2+}_p$		
x_{15}	RGS_a	x_{35}	$\mathrm{PKC} \cdot \mathrm{DAG} \cdot \mathrm{Ca}^{2+} \cdot \mathrm{PLC}\beta3 \cdot \mathrm{Ca}^{2+}$		
x_{16}	$\mathrm{RGS}_a \cdot \mathrm{G}\alpha_i\mathrm{GTP}$	x_{36}	$\mathrm{PLC}\beta_3 \cdot ca^{2+}_p$		
x_{17}	$\mathrm{RGS}_a \cdot \mathrm{G}\alpha_q\mathrm{GTP}$	x_{37}	$\mathrm{IP3}R$		
x_{18}	$\mathrm{PLC}\beta4$	x_{38}	$\mathrm{IP3R} \cdot \mathrm{IP3}$		
x_{19}	Ca^{2+}	x_{39}	$\mathrm{IP3R} \cdot \mathrm{IP3} \cdot \mathrm{Ca}^{2+}$		
x_{20}	$\mathrm{PLC}\beta4 \cdot \mathrm{Ca}^{2+}$	x_{40}	$\mathrm{IP3R} \cdot \mathrm{Ca}^{2+}$		

value is observed. Shown in Fig. S9B is the response of MUP to the same signal: x_{19} reaches a very high value in a short time and stays there without being brought down to the equilibrium value achieved by the full network. So, the feedbacks that our procedure has identified act as a controller to set up the output of the system to a controlled value, over a longer time-scale than that over which the MPU responds to external signals with fast production. This indicates similar type of dynamics taking place in this network as in other examples.

5 λ phage decision circuit

When the bacterium *Esherichia Coli* is infected by phage λ, there are two possible subsequent pathways: lysogeny and lysis depending on the concentration of particular proteins. In the lysogenic pathway, the DNA of the λ-phage will be integrated to the host DNA. In the lytic pathway, the host DNA will be excised to provide material for λ-phage duplication. The selection of the pathway is made by the λ lysis-lysogeny decision circuit where the molecular fluctuations play significant roles [9].

Here, we present a circuit model due to H. H. McAdams and L. Shapiro [10].

The decision between lysis and lysogeny described above is made by a well-characterized competitive regulatory mechanism of a bistable gene switch. The core of the switch is the P_R and P_{RM} operators which share three operator sites (OR1, OR2, OR3). Translation of P_R transcript produces CI while P_{RM} encodes *Cro.* The two factors CI and Cro bind to the operators sequentially in the opposite order. When CI binds to OR1, P_R is repressed, following which P_{RM} is activated by CI at OR2 and repressed by CI at OR3. P_{RM} is repressed when Cro binds to OR3 and P_R is repressed by Cro at OR2

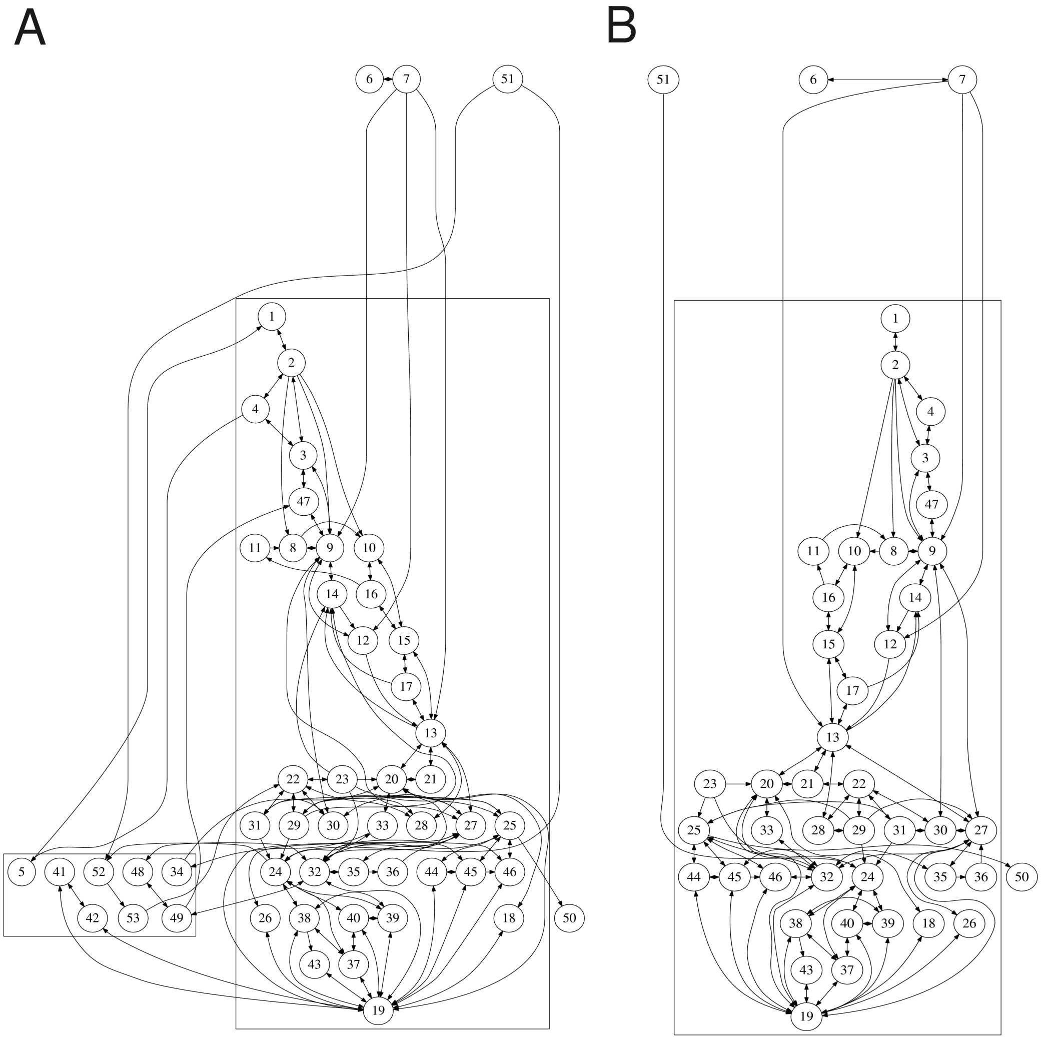

Figure S8: Graph analysis of the G-protein coupled receptor network. (A) Feedback and forward structure through graph decomposition. (B) The minimal production unit of the network.

or OR1. Initially P_{RM} is off. But CII initiated from P_R and CIII, N initiated from P_L lead to the stimulation of P_{RE}, which induces rapid production of CI and suppresses the production of Cro by the anti-Cro transcript. Both Cro and CI suppress P_L. Protein N is able to anti-terminate the terminators tR1, tR2, tL1, tL2, such that CII and CIII could be produced rapidly and the production of proteins Q, Xis, Int is able to start. Regulator Q can anti-terminate tR', which paves the way for the $P_{R'}$ initiated transcription to continue through S and activate the lysis coding genes. However, Q may be repressed by Anti-Q initiated from P_{AQ} which is activated by CII. The concentrations of Xis and Int will determine lysis-oriented excision or lysogenesis-oriented integration, respectively. The CII activated promoter P_I favors the production of Int.

In all, roughly speaking, CI, CII and CIII production leads to lysogenesis while Cro production leads to lysis. Both the bacteria state such as the multiplicity of infection (MOI) and external cues such as ultraviolet light play important roles in the fate decision process. Many components of the network are effective only transiently. Once the decision has been made, they are suppressed. In the model shown in Fig. S10, the

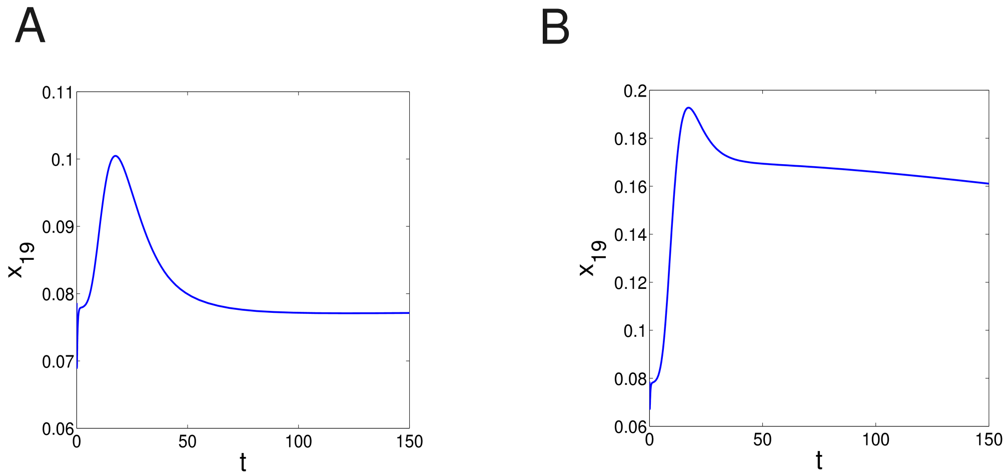

Figure S9: The evolution of the Ca^{2+} (x_{19}). (A) With all the feedbacks present and (B) only with MPU.

Table S4: In the table, P_{RM},P_R,P_{RE},P_L,P_I, P_{AQ},$P_{R'}$ are promoters; tR1, tR2, tL1, tL2, tR' are terminator on the DNA strand; CI, CII, CIII, N, O, P, Q, Cro, Xis, Int, Anti-Q are regulatory proteins; Anti-Q, Ant-Cro are gene transcripts. The corresponding gene is marked by lower case letters.

x_1	RecA	x_{11}	Cro	x_{21}	tL2	x_{31}	Anti − Q
x_2	ci	x_{12}	ant − Cro	x_{22}	tR2	x_{32}	$P_{R'}$
x_3	CI	x_{13}	Ant − Cro	x_{23}	tR2	x_{33}	tR′
x_4	CI∗	x_{14}	P_{RM}	x_{24}	o	x_{34}	P_I
x_5	cii	x_{15}	P_R	x_{25}	O	x_{35}	xis
x_6	CII	x_{16}	P_{RE}	x_{26}	p	x_{36}	Xis
x_7	CII∗	x_{17}	P_L	x_{27}	P	x_{37}	int
x_8	ciii	x_{18}	n	x_{28}	q	x_{38}	Int
x_9	CIII	x_{19}	N	x_{29}	Q	x_{39}	S
x_{10}	cro	x_{20}	tL1	x_{30}	anti − Q		

operators OR1, OR2, OR3 are not represented explicitly.

This network is quite sparse. x_1 represents the external signal source and sits on the top of the decomposed structure in Fig. S11A. Five elements $\{x_{25}, x_{27}, x_{36}, x_{38}, x_{39}\}$ stand at the bottom. in which $\{x_{25}, x_{27}\}$ are intermediate products that are not active in the model while the rest are important for the λ lysis-lysogen fate decision. The main processing part is contained in the left long bar where the signal enters through cii (x_5) and finally switches on (off) N (x_{19}) and tR1 (x_{22}). All the subsequent steps depend on these two. The feedback vertices are contained in the small vertical box to the right of the main processing unit. The Cro (x_{11}) autoregulation induced by gene transcription via $x_1 0$ and the CIII factor (x_9) are treated as feedbacks, which seems plausible. Also noticeable is the small loop in the main unit consisting of $\{x_{14}, x_2, x_3, x_4\}$ with one feedback from x_4 to x_{14}. This loop is just the autoregulation module of CI and can be identified if we applied a hierarchical version of our procedure. After removing the identified feedbacks, the MPU is obtained and displayed in Fig. S11B where the signal relaying process is very clearly exhibited. As before, the MPU is responsible for production of the response,

and the feedback box for its robustness and level.

References

[1] K. Fujarewicz, M. Kimmel, and A. Swierniak (2005) On fitting of mathematical models of cell signaling pathways using adjoint systems. *Math Biosci Engr* 2(3):527–534.

[2] C. V. Rao, J. R. Kirby, and A. P. Arkin (2004) Design and diversity in bacterial chemotaxix: a comparative study in *escherichia coli* and *bacillus subtilis*. *PLoS Biol* 2:0239–0252.

[3] P. Rangamani and L. Sirovich (2006) Survival and apoptotic pathways initiated by TNF-α: modeling and predictions. *Biotech Bioengr* 97:1216–1229.

[4] R. S. Kuczenski, K. C. Hong, J. García-Ojalvo, and K. H. Lee (2007) PERIOD-TIMELESS interval timer may require an additional feedback loop. *PLoS Comput Biol* 3:1468–1476.

[5] J. Schlessinger (2000) Cell signaling by receptor tyrosine kinases. *Cell* 103:211–225.

[6] H. Resat, J. A. Ewald, D. A. Dixon, and H. S. Wiley (2003) An integrated model of epidermal growth factor receptor trafficking and signal transduction. *Biophys J* 85:730–743.

[7] B. N. Kholodenko, O. V. Demin, G. Moehren, and J. B. Hoek (1999) Quantification of short term signaling by the epidermal growth factor receptor. *J Biol Chem* 274:30169–30181.

[8] P. J. Flaherty (2007) *A Kinetic Model for G protein-coupled Signal Transduction in Macrophage Cells* (PhD thesis, EECS Department, University of California, Berkeley).

[9] A. Arkin, J. Ross, and H. H. McAdams (1998) Stochastic kinetic analysis of developmental pathway bifurcation in phage λ-infected *escherichia-coli* cells. *Genetics* 149:1633–1648.

[10] H. H. McAdams and L. Shapiro (1995) Circuit simulation of genetic networks. *Science* 2669:650–656.

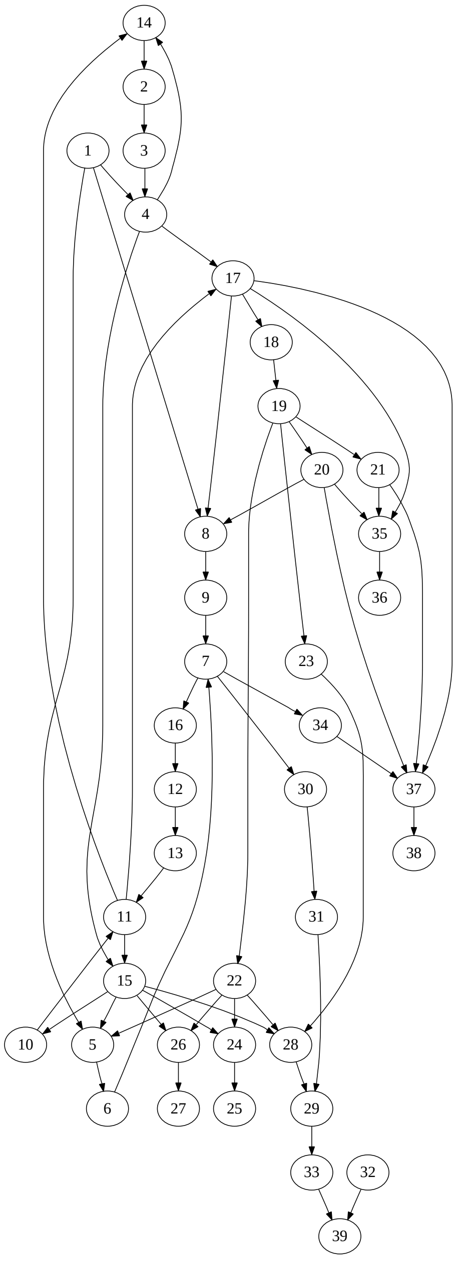

Figure S10: Network representation of the λ phage decision circuit.

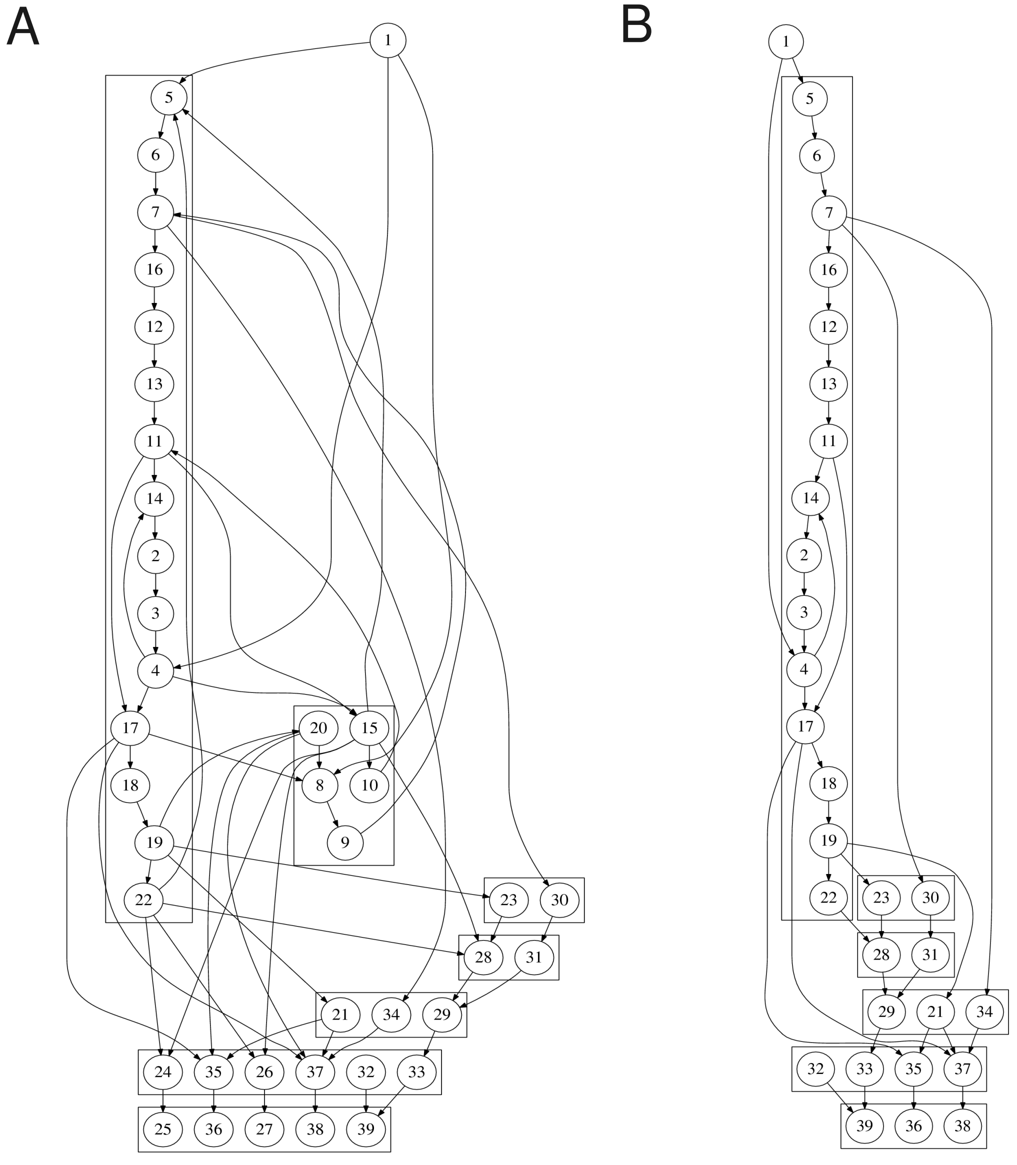

Figure S11: The graph analysis of the λ lysis-lysogen decision network. (A) Feedback and forward structure through graph decomposition. (B) The minimal production unit of the network.
